# Polymerization Assisted by Upconversion Nanoparticles under NIR Light

**DOI:** 10.3390/molecules24132476

**Published:** 2019-07-05

**Authors:** Polina Demina, Natalya Arkharova, Ilya Asharchuk, Kirill Khaydukov, Denis Karimov, Vasilina Rocheva, Andrey Nechaev, Yuriy Grigoriev, Alla Generalova, Evgeny Khaydukov

**Affiliations:** 1 Federal Scientific Research Center «Crystallography and Photonics» Russian Academy of Sciences, Leninskiy Prospekt 59, Moscow 119333, Russia; 2 Shemyakin-Ovchinnikov Institute of Bioorganic Chemistry Russian Academy of Sciences, Miklukho-Maklaya str. 16/10, Moscow 117997, Russia; 3 Institute of Fine Chemical Technologies, Moscow Technological University, Vernadsky Avenue 78, Moscow 119454, Russia; 4 I.M. Sechenov First Moscow State Medical University, Trubetskaya str. 8-2, Moscow 119991, Russia; 5 Institute of Mathematics and Informational Technologies, Volgograd State University, Universitetskiy Prospect, 100, Volgograd 400062, Russia

**Keywords:** photopolymerization, nanocomposite materials, upconversion nanoparticles, NIR light, surface modification

## Abstract

Photopolymerization of nanocomposite materials using near infrared light is one of the unique technologies based on the luminescent properties of lanthanide-doped upconversion nanoparticles (UCNPs). We explored the UCNP-triggered radical polymerization both in oligomer bulk and on the nanoparticle surface in aqueous dispersion. Core/shell UCNPs NaYF_4_:Yb^3+^ and Tm^3+^/NaYF_4_ with emitting lines in the ultraviolet and blue regions were used to activate a photoinitiator. The study of the bulk photopolymerization in an initially homogeneous reaction mixture showed the UCNP redistribution due to gradient density occurring in the volume, which led to formation of UCNP superlattices and spheres “frozen” in a polymer matrix. We also developed a strategy of “grafting from” the surface, providing polymer shell growth directly on the nanoparticles. The photosensitization of the endogenous water-soluble photoinitiator riboflavin by the resonance energy transfer from UCNPs was demonstrated in the course of monomer glycidyl methacrylate polymerization followed by photocrosslinking with poly(ethylene glycol) diacrylate on the nanoparticle surface.

## 1. Introduction

Lanthanide-based upconversion nanoparticles (UCNPs) are a promising nanoplatform for future technologies. UCNPs are a class of photoluminescent material, which have attracted much attention due to their ability to convert low-energy near-infrared (NIR) light into visible and UV photons in the course of the sequential absorption of two or more quanta [1]. UCNPs consist of an inorganic crystalline host matrix, co-doped with a pair of trivalent lanthanide ions (Ln^3+^), usually with Yb^3+^ (as a sensitizer) and Er^3+^ or Tm^3+^ (as an activator) [2]. NIR light excitation, unlike UV and visible light [3], possesses an ability for deep penetration into biotissues with no photodamage to living cells, leading to the rapid progress of NIR technology in biomedicine. Furthermore, UCNPs are free of the typical drawbacks of conventional fluorophores (quantum dots, nanodiamonds, fluorescence proteins, dyes, etc.) [4,5], and demonstrate high photostability; a lack of blinking and bleaching; narrow emission lines and a large anti-Stokes shift; and low cytotoxicity. All these peculiarities show a highly versatile and translatable UCNP photoluminescent nanotechnology for application in industry and life science [6].

Three-dimensional prototyping of nanocomposite photopolymer materials using NIR light is one of the unique technologies based on the luminescent properties of UCNPs. This process is similar to the process of two photon polymerization (2PP), but at the same time devoid of its shortcomings, such as a long curing process due to the small two-photon absorption cross-sections of used chromophores [7] and, more importantly, the use of a high intensity laser. Unlike the 2PP process, UCNPs can be excited by using semiconductor continuous-wave lasers with relatively moderate intensity, usually 10 W/cm^2^ [8]. In addition, this technology inherits all the advantages of 2PP, including the NIR light that falls in the “optical window” with minimal absorption and scattering of typically used monomers and polymers [9]. Under NIR radiation UCNPs can play the role of nanolamps, emitting light deeply inside the composition, which is exploited in order to initiate the photopolymerization of various monomers [10,11,12,13,14,15,16] and photocurable materials [17], and to produce 3D objects directly in the bulk of light-sensitive resins [18,19,20]. Although the NIR photopolymerization strategy is simple and has already been shown, there is still not enough knowledge about the mechanism of the polymerization initiation, polymer chain propagation, distribution of nanoparticles in the composition, and spatial resolution, etc.

NIR light-activated polymerization triggered by UCNPs also gives a new opportunity in the field of nanoparticle surface modification, which is crucial for its biomedical applications. This strategy offers a perspective approach for polymer-shell formation through the chain growth from the UCNP surface. Overlapping the UCNP emission and photoinitiator absorption spectra leads to a polymer shell with controlled thickness attached tightly to UCNP surface, forming a dense layer under NIR irradiation in the dispersion medium, preventing nucleation of new particles. In addition, this approach allows for the embedding of drugs during polymer shell formation on the UCNP surface. To date, only a few works demonstrated NIR-activated surface modification [13,21,22,23]. Bagheri et al. demonstrated that highly efficient RAFT-polymerization can be used to grow polymer chains directly on the surface of inorganic materials (“grafting from”) such as lanthanide doped UCNPs [21]. This polymerization process allowed for production of aqueous nanoparticle dispersions with controlled density and thickness of the polymer shells. Despite all of the advantages (e.g., retaining UCNP optical properties, colloidal stability after polymer shell formation), the RAFT-polymerization is a very complicated process that requires highly-skilled labor and has the potential for hydrolysis of the RAFT-initiator. Xiao et al. demonstrated NIR light-activated photopolymerization of polyethyleneglycol-diacrylate (PEG-DA) on the UCNP NaYF_4_:Er^3+^,Yb^3+^ surface in the presence of Eosine Y [22]. However, UCNPs tend to aggregate from a uniform dispersion (100–200 nm) to nanoclusters (3–10 mm), while the irradiation time of the NIR light increases. The use of Eosin Y in highly sensitive formulations requires the exclusion of day light or at least the use of special safety light [23]. Beyazit et al. employed oleic acid (OA)-capped UCNPs as an internal light source for photopolymerization in situ of a hydrophilic monomer 2-hydroxyethyl methacrylate (HEMA) and the crosslinker N,N′-ethylenebis- (acrylamide) (EbAM) to obtain a thin polymer shell around the nanoparticles [13]. UCNPs were employed to activate the UV (benzophenone/triethylamine) and visible (eosin Y/triethylamine) initiators and obtain colloidally stable particles of about 50 nm in diameter. However, this technique requires a long period of exposure (4 h). Thus, the development of an approach for a technological polymer shell formation on the UCNPs surface is crucial. 

Herein, we report a facile strategy of UCNP-triggered radical polymerization under NIR irradiation both in monomer (oligomer) bulk and on the UCNP surface. We studied bulk polymerization under NIR irradiation in more detail, and showed that, unlike our previous results presented in [19], the utilization of a UCNP concentration below the percolation threshold in a reaction mixture resulted in UCNP redistribution in the reaction volume in the form of superlattices and spheres “frozen” in polymer. We also developed a strategy of “grafting from” the surface, providing polymer shell growth directly on the UCNP under NIR light. The potential of the endogenous water-soluble photoinitiator riboflavin was shown in the course of monomer glycidyl methacrylate (GMA) polymerization followed by photocrosslinking with polyethyleneglycol-diacrylate (PEG-DA) on the surface of UCNPs–GMA. We believe that this quick strategy enables an effective surface modification of nanoparticles, expanding the application of UCNP for industry and biomedicine.

## 2. Results and Discussion

**Figure 1:**
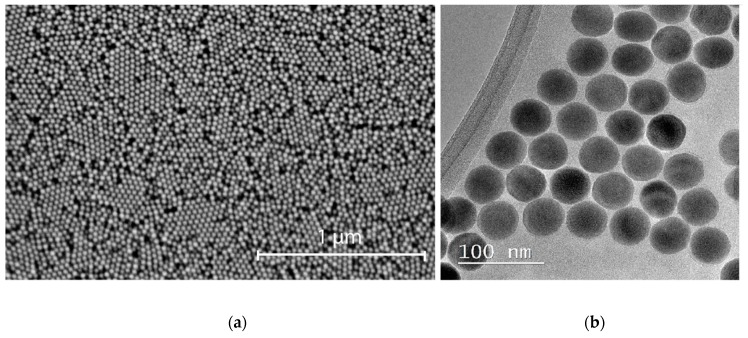
(**a**) SEM and (**b**) TEM images of core/shell NaYF_4_: Yb^3+^, Tm^3+^/NaYF_4_ nanoparticles.

We used UCNPs β-NaYF_4_:Yb^3+^, Tm^3+^ with a core/shell structure, synthesized by a modified solvothermal method, as described in [24]. Nanoparticle size was evaluated by TEM as 40 nm × 30 nm (Figure 1a,b) with a hexagonal (*P6_3_/m* space group) crystal structure favorable for the energy-transfer upconversion process. 

**Figure 2:**
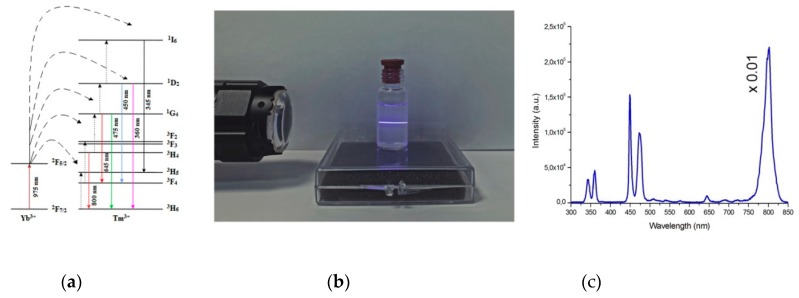
(**a**) Energy level diagram of upconversion nanoparticles (UCNPs): NaYF_4_: Yb^3+^, Tm^3+^; (**b**) Visible spectral range image of UCNPs photoluminescence in hexane; (**c**) Corrected apparatus function spectrum of the UCNPs under near-infrared (NIR) excitation at 975 nm at intensity 10 W/cm^2^ (line centered at 800 nm multiplied by coefficient 0,01).

The upconversion mechanism is based on the nonlinear conversion of NIR light through real energy states of trivalent lanthanide ions (see Figure 2a) [25]. Ion Yb^3+^ in the UCNPs absorbs a 975 nm photon and is excited to the ^2^F_5/2_ state; then, it transfers its energy to the ^3^H_5_ state of a neighboring Tm^3+^. Thereafter, an additional energy transfer occurs from another excited Yb^3+^ to the Tm^3+^, resulting in further excitation to a higher level of Tm^3+^. The excited Tm^3+^ is able to emit upconverted photons (ca. 345, 360, 450, 475, 645, or 800 nm) with higher energy levels than those of the exciting photons. As a result, the intensities of the UCNP photoluminescence lines nonlinearly depend on the intensity of the incident NIR light. The general rule can be written as

(1) $$I_{em}\;\sim \;{\left[I_{ex}\right]}^{n}$$

where n is the number of NIR quanta involved in the upconversion process [26].

The photoluminescence spectrum (Figure 2b) of the synthesized UCNPs had strong lines centered on NIR (800 nm), red (645 nm), blue (475, 450 nm), and UV (345, 360 nm) spectral regions under excitation at a wavelength of 975 nm at intensity 10 W/cm^2^, which corresponds to 4f transitions of Tm^3+^ ions. Measurements of samples using an integrating sphere showed that nanoparticles had high integral conversion efficiency (~9%) at the excitation intensity 30 W/cm^2^.

### 2.1. UCNP-Assisted NIR Polymerization in Bulk

In this section, we demonstrate UCNP-assisted photopolymerization of oligocarbonate methacrylate (OCM-2) in the presence of photoinitiator Irgacure 369 under NIR irradiation. In order to start photopolymerization, the initiator should be activated by UCNP light. UCNP wavelengths at 345 and 360 nm fall within the absorption band of the chosen photoinitiator. UCNPs convert near infrared light into UV photons that activate the photoinitiator and start the polymerization process. In our work [19], we explored three-dimensional (3D) rapid prototyping technology based on near-infrared light-induced polymerization of photocurable compositions containing upconversion nanomaterials. We showed that the evaluation of the UCNP concentration threshold in a photocurable composition is required for 3D structure formation due to different polymer growth kinetics on the flat and the narrow edge surfaces of single UCNPs. This study led us to the conclusion that the threshold of UCNP concentration during polymerization should exceed 16.8 mg mL^−1^ in photosensitive compositions for 3D structure formation with high spatial resolution. This phenomenon is explained by the mathematical model of percolation, which has a geometrical-statistical character, for details see [19]. In this work, we focus on the polymerization of photosensitive resin, which contains an order of magnitude lower concentration of UCNPs, namely below the percolation threshold. The rate of UCNP-assisted polymerization non-linearly depends on the NIR light intensity, due to the non-linear nature of the upconversion process. In addition, changing the concentration of nanoparticles in a photocomposition is necessary to maintain the total dose of upconverted UV photons required for the polymerization reaction, which in the first approximation depends linearly on the concentration of nanoparticles in the photocomposition.

**Figure 3:**
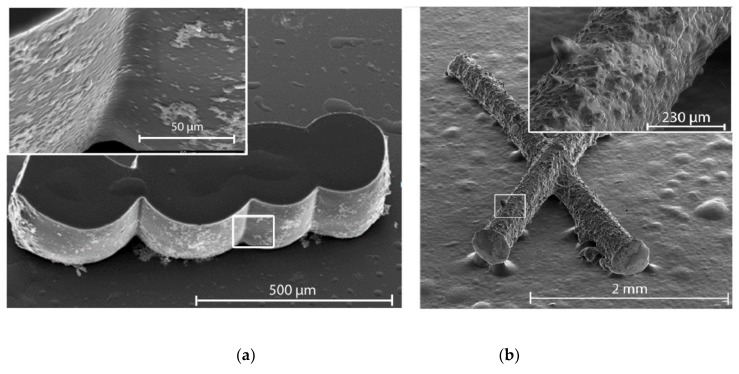
SEM image of 3D polymer microstructures obtained by NIR light-activated photopolymerization: (**a**) UCNPs concentration ~ 20 mg mL^−1^ and (**b**) UCNPs concentration ~ 2 mg mL^−1^.

Figure 3 shows the SEM-images of a UCNP structure obtained in the course of polymerization. The image on the left side (Figure 3a) demonstrates the structures obtained in the same regime as the structures described in [19] at a nanoparticle concentration of ~ 20 mg mL^−1^. In the right image (Figure 3b), the composition was photopolymerized at a nanoparticle concentration of ~ 2 mg mL^−1^. The use of any UCNP concentrations led to the structure formation in NIR irradiated volume. However, with higher magnification (inset), a significant difference in the roughness, despite an initially homogenous chemical photocomposition of the sample, was observed. We believe that this effect is associated with the peculiarities of the photopolymerization process. Since the process of NIR-induced polymerization with UCNPs above the percolation threshold was studied in detail earlier in [19], here we present the structure images in Figure 3a only for comparison of the structure surface morphology. It would be reasonable to assume that UCNP concentration below the threshold results in structures in the form of nanoparticles with a polymer shell. The thickness of the shells may be determined by the mean photon-free path in the photosensitive composition,

(2) $$I_{pl}=\frac{1}{\alpha_{pl}}=\frac{1}{\varepsilon_{pl}\times C_{pl}}$$

where *α_pl_, ε_pl_,* and *C_pl_* denote the linear absorption coefficient, molar extinction coefficient, and molar concentration of the photoinitiator, respectively. At a sufficient UV photon flux from the UCNPs and taken that the mean photon-free path in the medium exceeds half of the mean distance between the UCNPs, the polymer shells formed around UCNPs start to interact, leading to a bounded volume. However, in this context, we did not take into account the fluctuation of volumes with different polymer density, which in turn are responsible for the nanoparticle movement during photopolymerization. Nanoparticles are known to diffuse from the illuminated areas into the dark fields under the condition of spatially inhomogeneous illumination of a nanocomposite material. This effect appears as a result of density gradients and, for example, can be used to write phase gratings in a nanocomposite polymer material [25].

**Figure 4:**
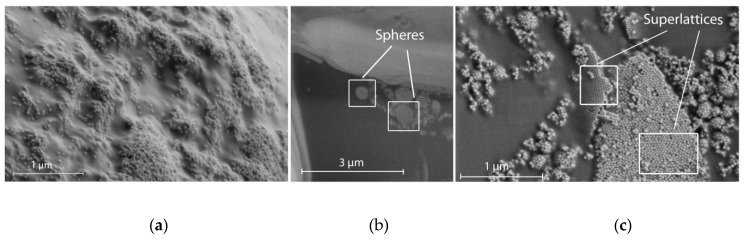
(**a**) SEM photograph of the polymer structure region with a disordered distribution of UCNPs. Two types of ordered nanoparticle structures formed in the nanocomposite in the course of photopolymerization: spheres (**b**) and superlattices (**c**).

A detailed examination of the structure surface and sections showed that two types of ordered nanoparticle structures distributed randomly can be formed in nanocomposites in the course of photopolymerization: superlattices and spheres (see Figure 4). Formation of superlattices probably occurs due to the frontal polymerization [26] associated with a traveling wave developed under light action in a certain area of photocomposition at the microlevel. Photopolymerization results in a density increase of the photocomposition, providing a density gradient along one of three coordinates. A wave moving along the photocomposition rearranges the embedded nanoparticles to the wave boundary and, at the one-dimensional wave propagation they form tightly packed layers, which sooner or later are “frozen” into the polymer matrix (Figure 4b). Note that another scenario can take place. In the case of a three-dimensional counter-propagating wave (the case of compression), densely packed spherical agglomerates consisting of nanoparticles can be formed (Figure 4a). The analysis of the polymer structure sections made by an ion beam showed such spherical agglomerates of nanoparticles formed by NIR-induced polymerization. It is worth noting that these two processes are random and determined by the stochastic distribution of UCNPs in the composition bulk. 

### 2.2. Graft Surface Polymerization Onto UCNP in Dispersion Under NIR Irradiation

In this section, we consider NIR-induced polymerization in UCNP aqueous dispersion, not in the monomer (oligomer). Photopolymerization under NIR light is one of the challenging methods for UCNP modification and the design of hybrid structures for biomedical applications. Our task was to modify the UCNP surface with polymer shells produced under NIR light. Modification governs the formation of polymer shells comprised of biocompatible material, with controlled thickness, firmly attached to the surface without polymer particle nucleation in the dispersion medium. This approach requires low UCNP concentration and hydrophilization in order to ensure colloidal stability and growth of polymer chains from the surface for initiation of ‘‘grafting from” polymerization.

Only a few works addressed NIR-activated photopolymerization in the presence of UCNPs [13,21,22]. Typically, the use of hydrophilic UCNP nanoparticles in vivo, with modification under NIR light as described in the literature, is limited by exploitation of exogenous photoinitiators in the polymerization. There are a large number of photoinitiators characterized by water solubility, biocompatibility, and lack of a cytotoxic effect with 2,2′-azobis[2-methyl-N-(2-hydroxyethyl)propionamide] (VA-086), lithium phenyl-2,4,6-trimethylbenzoylphosphinate (LAP), Irgacure 2959 (1-[4-(2-hydroxyethoxy)-phenyl]-2-hydroxy-2-methyl-1-propane-1-one), Eosin-Y, and others [27]. However, only a few photoinitiators reported in the literature are utilized as complexes with UCNPs irradiated with NIR light (e.g., Eosin-Y, Riboflavin) [28,29,30]. Here, we applied the endogenous, FDA-approved, non-toxic compound riboflavin in its water-soluble form flavin mononucleotide (FMN) as the photoinitiator for polymerization. Under UV and visible light, FMN was able to produce active radicals in the presence of the coinitiator triethanolamine (TEOHA) [31], which activates polymerization.

We studied the NIR light-activated photopolymerization in the presence of UCNP concentrations under the percolation threshold. Low UCNP concentrations provide thin shell formation on the surface that prevent UCNP aggregation and nucleation of new polymer particles. In general, the polymerization design includes the UCNP surface hydrophilization with tetramethylammonium hydroxide (TMAH); modification with ethylene diamine (EDA), FMN, and glycidyl methacrylate (GMA); polyethylene glycol diacrylate (PEG-DA) immobilization onto the UCNP surface; followed by NIR light triggering for nanoparticle surface modification.

**Figure 5:**
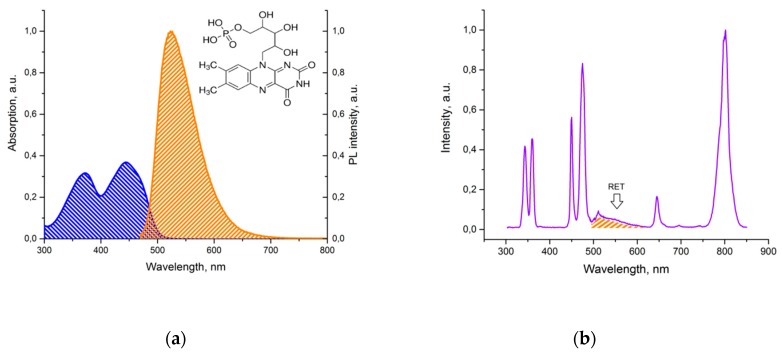
(**a**) The excitation (blue) and emission (orange) spectra of flavin mononucleotides (FMN), in insert: FMN formula; (**b**) Photoluminescence of UCNP–FMN in water under 975 nm excitation.

The modification process was carried out in several stages. In order to increase the affinity between the UCNP surface and both the monomer and photoinitiator, an excess of oleic acid (OA) was removed from the UCNP surface with TMAH, then the surface was stabilized by EDA, ensuring amine groups on the UCNP surface. OA partial removal makes the UCNP surface available to FMN molecules. UV and blue photoluminescent lines of UCNPs (Figure 2b) overlap the FMN absorption band (Figure 5b), thus enabling design of the energy transfer donor–acceptor pair UCNP–FMN, relying on resonance energy transfer processes (RET). To ensure an effective RET from the UCNP to the FMN molecule, the distance between donor–acceptor pair should be 3–10 nm as described earlier [24]. FMN coordinates the rare earth metal ions of the UCNPs owing to the presence of the PO_4_^−^ groups (Figure 5a) [32]. After this modification step, a broadband fluorescence signal from 500 to 625 nm, ascribed to the FMN emission under 975 nm excitation, manifested the energy transfer between UCNP and FMN (Figure 5b). The presence of amine groups on the UCNP surface allows for GMA immobilization at the next step and provides tight monomer GMA coupling and propagation of polymer chains from the UCNP surface.

**Figure 6:**
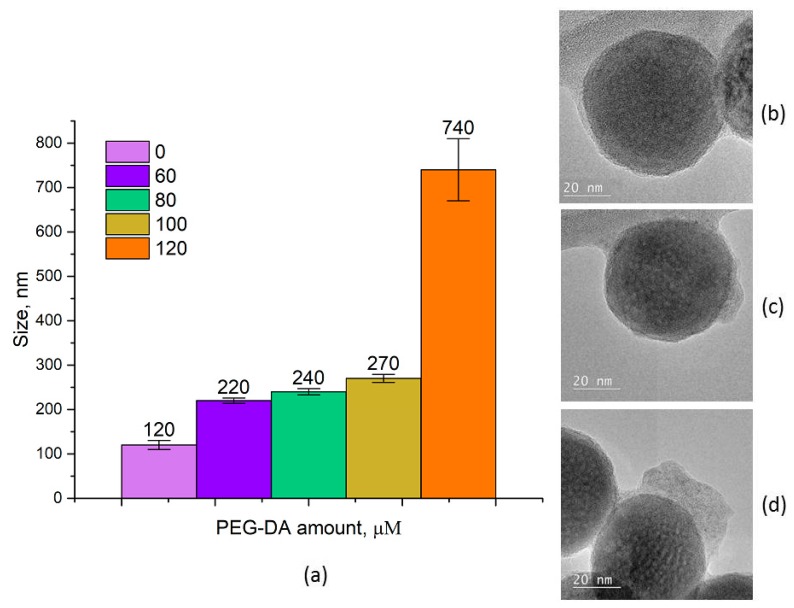
(**a**) Hydrodynamic sizes of UCNP–GMA–PEG via PEG-DA concentration, measured by dynamic light scattering; TEM images of nanoparticles: (**b**) UCNP–GMA; (**c**) UCNP–GMA–PEG, 60 μM PEG-DA; (**d**) UCNP–GMA–PEG, 100 μM PEG-DA. GMA = glycidyl methacrylate; PEG = polyethylene glycol; PEG-DA = polyethylene glycol diacrylate.

The non-toxic in vivo reagent should possess a biocompatible surface, for example, the same as the PEG molecule can impart. PEG-based materials offer unique biocompatibility and can be used for clinical applications [33,34]. Thus, the next step involved introduction of a hydrophilic biocompatible cross-linker PEG-DA into the system, followed by NIR irradiation to start polymerization GMA. The process was carried out in a scanning mode of irradiation at a laser power of 10 W/cm^2^ for 45 min. The UCNP–GMA–PEG sizes were evaluated by dynamic light scattering (DLS) (Figure 6a). The increase of cross-linker concentration led to growth of the UCNP diameters from 120 to 270 nm. At the maximal concentration of PEG-DA (120 μM), the agglomerate formation (740 nm) was observed. TEM images demonstrate that GMA polymerization resulted in a uniform, thin, polymer shell formation (Figure 6b), while the cross-linker addition provided the formation of a knobby polymer shell (Figure 6c,d) with an increase in the uniformity of the shell at higher concentrations of PEG-DA. The different TEM and DLS results can be explained by the hydrophilic nature of polymer coatings that shrink during sample preparation for TEM. In addition, the heterogeneity of the polymer coating that was observed was most likely due to diffusion, insufficient mixing, and the concentration gradient of the polymer inside the composition.

**Figure 7:**
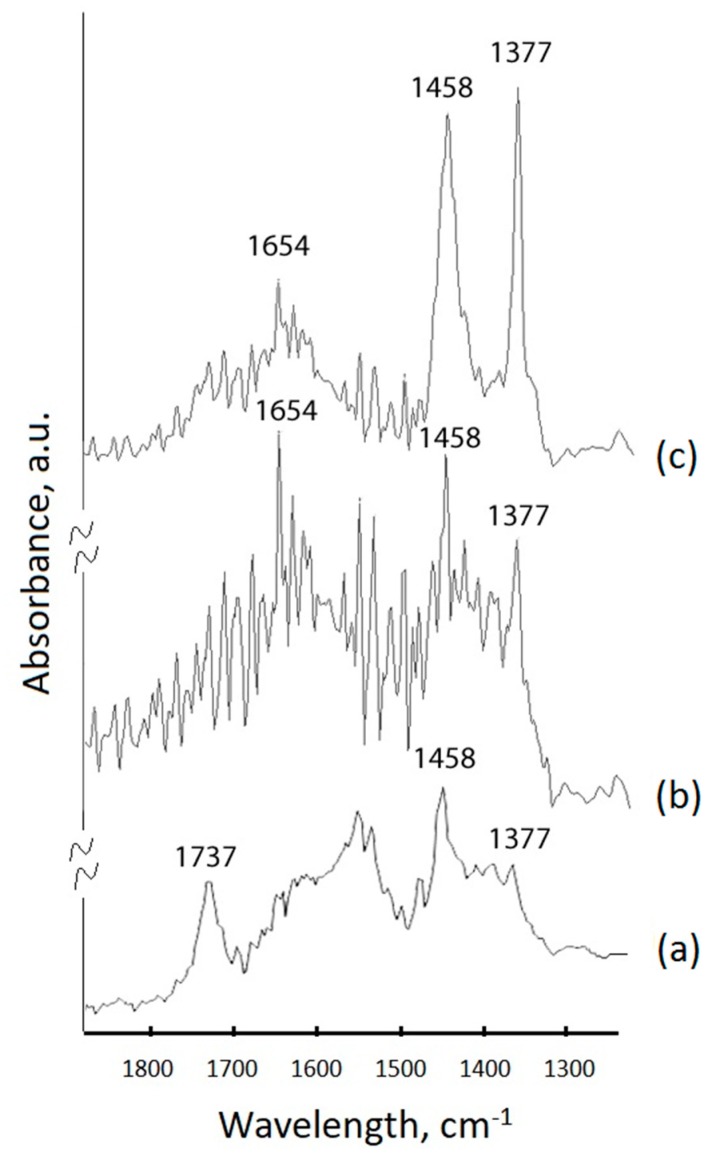
FTIR-spectra of the UCNP samples: (**a**) UCNPs hydrophilized with tetramethylammonium hydroxide (TMAH); (**b**) UCNP–GMA before photo-induced crosslinking; (**c**) UCNPs encapsulated in PEG-DA.

FTIR spectroscopy of the PEG surface-capped particles further confirmed the successful surface modification of the UCNP samples. The results of the comparative analysis of UCNP–TMAH, UCNP–GMA, and UCNP–PEG are presented in Figure 7. A strong stretching vibration of –C=O assigned to oleic acid appeared at 1737 cm^−1^ in the sample treated with TMAH (Figure 7a). However, this characteristic band almost disappeared in the sample of UCNP–GMA before photo-induced crosslinking and in the sample of UCNPs encapsulated in PEG-DA (Figure 7b,c). C–H stretching of the CH_2_ polymer groups at 1458 cm^−1^ and 1377 cm^−1^ appear in UCNP–GMA and increase in UCNP–PEG samples. The featured peak at 1654 cm^−1^, related to the carboxylic groups in UCNP–GMA, disappeared in the UCNP–PEG as a result of the polymerization.

The described method enables photopolymerization followed by cross-linking exclusively on the UCNP surface, eliminating polymer formation in the bulk of the composition and allowing for size control of the UCNP agents. Utilizing non-toxic riboflavin, also called vitamin B_2_, as a photoinitiator in combination with a PEG coating allowed for production of a stable aqueous nanoparticle bioprobe. Low radiation intensity, short irradiation time, and adaptability for a specific task are advantages for luminescent probe preparation; these qualites are able to solve optical imaging problems, for example, in passive tumor labeling, where PEG-modified upconversion nanoparticles have proven to be advantageous [35].

The proposed approaches can be adapted for a number of applications based on the delivery of initial liquid materials to the subsurface and confined spaces followed by solidification and shaping. UCNP-based UV emission under NIR light irradiation enables “delivery” of the irradiation to the photocomposition at certain depths. At the same time, the low intensity of the NIR light means this technique has the potential to be employed for various biomedical applications. For example, the first approach can be used for direct writing to prepare the macro- and microstructural configuration of various implants. The second approach can be useful for the UCNP hydrophilization and functionalization required for bioreagents used in biomedical analyzes and visualization.

## 3. Materials and Methods

### 3.1. Materials

The following materials were purchased from Sigma-Aldrich (USA), and used without further purification: oligocarbonate methacrylate (OCM-2), photoinitiator Irgacure 369, triethanolamine (TEOHA), dimethyl sulfoxide (DMSO), tetramethylammonium hydroxide (TMAH), ethylene diamine (EDA), glycidyl methacrylate (GMA), polyethylene glycol diacrylate (PEG-DA), Mm ~ 575, potassium bromide, hexane, and chloroform. Flavin mononucleotide (FMN) was purchased from Pharmstandart (Dolgoprudny, Russia).

### 3.2. Methods

Absorbance characteristics were measured using a Cary 50 (Varian, Palo Alto, CA, USA). Luminescence spectra were recorded by a spectrofuorometer Fluorolog 3 (Horiba Jobin Yvon, France). The conversion efficiency of the nanoparticles was measured by an integrating sphere (Labsphere, North Sutton, NH, USA). FTIR spectra were recorded using an FTIR spectrophotometer (Varian 3100, Palo Alto, CA, USA). The TEM images of the UCNPs were obtained on a transmission electron microscope FEI Osiris (FEI, ‎Hillsboro, OR‎, USA). Morphology of the nanocomposite polymer samples were investigated by a scanning electron microscope Scios (FEI, Hillsboro, OR‎, USA) at 1 kV. Cross-sections of the polymer samples were made by a focused Ga+ ion beam technique at 30 kV accelerating voltage. Particle sizes were measured using a particle analyzer T90Plus (Brookhaven Corporation Instruments, Holtsville, NY, USA).

#### 3.2.1. UCNP-Assisted NIR Polymerization in Bulk

A mixure of oligocarbonate methacrylate (OCM-2), photoinitiator Irgacure 369, and nanoparticles was used as the photocurable composition for the UCNP-assisted NIR polymerization in bulk. First, 10 mg of Irgacure 369 was mixed with 1 g OCM-2, then, 100 μL UCNP dispersed in hexane (c = 0.2 g/mL) was added and the mixture was thoroughly shaken and sonicated until the hexane evaporated. The photopolymerization occurred in a laser beam (975 nm) focused into a specific volume of glass vial containing the photocurable composition at a laser power of ~ 100 W/cm^2^ for 20 s.

#### 3.2.2. Graft Surface Polymerization of UCNP in Dispersion Under NIR Irradiation

A volume of 40 μL UCNP dispersed (20 mg mL^−1^) in chloroform was added to 1 mL of a 3% aqueous solution of TMAH, thoroughly shaken, and sonicated until chloroform evaporated. The obtained probes were purified with centrifugation at 13,400 rpm for 10 min. The supernatant was replaced with distilled water. The obtained probes were stabilized with ethylene diamine (EDA) by incubation in an EDA 0.3% solution for 24 h.

To obtain UCNP–FMN nanocomplexes, UCNP–EDA nanosystems (c=0.8 mg ml^−1^) were incubated with an FMN solution (c = 10 mg mL^−1^) for 30 min with constant stirring at room temperature. Non-included FMN were removed by centrifugation at 13,400 rpm for 10 min. The supernatant was replaced with distilled water. The procedure for centrifuging and replacing the supernatant was repeated three times. As a result, hydrophilic UCNP–FMN nanocomplexes with effective energy transfer from nanoparticles to FMN molecules were observed.

The presence of amine groups on the UCNP surface allowed for GMA immobilization at the next step. Aqueous media UCNP–FMN was replaced with dimethyl sulfoxide (DMSO) and 20 μL of GMA was added to the DMSO dispersion of UCN–PFMN and incubated for 24 h. Just ahead of the polymerization process, 60, 80, 100, or 120 μM of cross-linking agent polyethylene glycol diacrylate (PEG-DA) and 2 μL triethanolamine (TEOHA) (c = 1 mg mL^−1^) were added to the photocurable composition. The polymerization was carried out in a scanning mode of irradiation at a laser power of 20 W/cm^2^ for 45 min. The resulting probes were purified with centrifugation at 13,400 rpm for 10 min. As a result, hydrophilic UCNP–PEG nanocomplexes were observed.

#### 3.2.3. Fourier-Transform Infrared (FTIR) Spectroscopy

Pure UCNP–PEG were thoroughly grounded and then pressed with KBr to form a tablet. The samples of UCNP were modified with PEG-DA at 60 and 100 nmol and were further modified with GMA prepared as potassium bromide (KBr) pellets. FTIR spectra were recorded using an FTIR spectrophotometer (Varian 3100, Palo Alto, CA, USA).

## 4. Conclusions

We have developed two approaches of NIR light-activated polymerization triggered by UCNPs. The first approach based on NIR-induced polymerization of oligomers in bulk in the presence of a typical photoinitiator resulted in the formation of a polymer structure with embedded superlattices and spherical aggregates. This process is associated with two mechanisms of polymerization under NIR irradiation: 1. due to the initiator activation generating radicals as a result of UCNP-induced conversion of NIR light into UV photons; 2. due to the frontal polymerization at a microscopic level as a result of the polymer density gradient. The second approach was based on polymer shell formation on the UCNP surface in an aqueous dispersion medium. The sensitization of the photoinitiator under NIR irradiation by the resonance energy transfer from UCNPs was achieved with the aim of demonstrating the great potential of the biocompatible, non-toxic endogenous reagent FMN for the production of PEG-modified UCNPs that are in high demand for industry and biomedical applications.
